# Ideal Soft Tissue Facial Profile in Iranian Males and Females: Clinical Implications

**Published:** 2018-05

**Authors:** Amir Ali Mafi, Reza Shahverdiani, Parviz Mafi

**Affiliations:** 1 Clinical Research and Development Center, Shahid Beheshti University of Medical Sciences, Tehran, Iran; 2 Department of Plastic Surgery, Shahid Beheshti University of Medical Science, Tehran, Iran

**Keywords:** Soft tissue, Facial profile, Caucasian, Aesthetic surgery

## Abstract

**BACKGROUND:**

Proper pre-operative facial analysis that includes a thorough evaluation of both the bony and soft tissue anatomy is paramount to success in performing aesthetic surgery of the face. Ethnic variations in soft tissue profile add an important variable to pre-operative facial analysis. The aim of our study was to determine the role of ethnic variations in soft tissue facial profiles through profile analysis of Iranian male and female patients.

**METHODS:**

Photographs of 100 Iranian males and 100 Iranian females (16 to 40 years old) were carried out. A review committee selected 10 male images and 10 female images, which they believed to be most ideal. The soft tissue profiles were then analyzed. A total of 21 measurements were analyzed and statistically compared with North American Caucasian males and females.

**RESULTS:**

The upper lip projection and lower lip projection were significantly more prominent in Iranian males as compared with North American Caucasian males. In addition, Iranian males had longer face as compared with North American Caucasian males along with a more drooping nasal tip. The frontonasal area is straighter and the lower face is longer in Iranian females compared with North American Caucasian in addition to more convex faces along with a shorter upper face.

**CONCLUSION:**

Significant differences in ideal soft tissue profiles exist between Iranian and Caucasian males. These differences should be recognized as they may play an important role in performing facial aesthetic and reconstructive procedures, particularly rhinoplasty, genioplasty, midface/facelifts, lip augmentation, and maxillofacial surgery.

## INTRODUCTION

Although the underlying skeleton defines the shape and size of the face, the overlying soft tissue is as important as the skeleton in facial appearance.^1^ Ideal facial profiles have been studied in medicine and art by Ricketts.^2^ Attention has also been given to details about the morphological and proportional upper, middle, and lower thirds of the face.^3^^-^^6^ The frontonasal angle, columellar-lip angle, lip-chin relationship, nose-chin-lip relationships,^6^ projection of the chin and maxilla in relation to the facial plane have all been described as important parameters when evaluating the face for cosmetic procedures.^3^^,^^5^^,^^6^

In particular, rhinoplasty, genioplasty, and lip augmentation procedures, require detailed knowledge of the normative values of the specific ethnic subgroup that is to be operated on. In addition, the importance of proper, individual pre-operative evaluation cannot be over-emphasized. In order to properly treat congenital or post-traumatic facial disfigurements, surgeons may benefit from access to facial profile databases for specific ethnic populations, that are based on accurate anthropometric and morphologic measurements.^2^^-^^19^

Previously, a comparison of these databases with the established norms of North American Caucasians has offered a suitable way to select a method for successful treatment.^1^ The purpose of this study is to analyze and describe the ideal aesthetic facial profile in Iranian males and females. This profile analysis may assist plastic surgeons who want to perform aesthetic and reconstructive surgery on Iranian and Middle Eastern faces and may help decrease the risk of creating post-operative “racial incongruity”. 

## MATERIAL AND METHODS

This study was carried out on 200 photographs of Iranian males (n=100) and females (n=100). None of the study patients had noticeable facial disfigurements or trauma. The age of the subjects ranged from 16 to 40 years and no significant differences were seen between male or female age ranges. Each photograph was scanned, the image was projected onto a computer monitor, and computerized sketches were obtained based on the photographs.

**Fig. 1:**
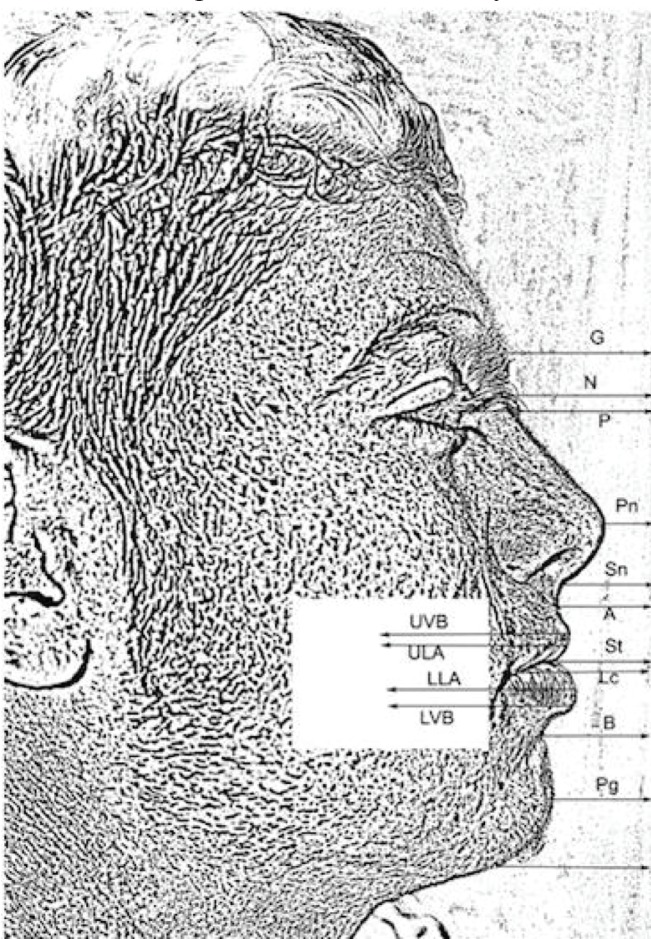
Soft tissue landmarks on facial profile. Soft tissue glabella (G’): most prominent or anterior point in the mid sagittal plane of the forehead.^7^ Soft tissue nasion (N’): The most concave point of the tissue overlying the area of the frontonasal suture.^7^ Pupil (P): The most anterior point in the midsagittal plane of the lens of the eye.^7^ Pronasale (Pn): The most prominent or anterior point on the midsagittal profile of the nose.^8^ Subnasale (SN): A point located at the junction between the lower border of the nose and the beginning of the upper lip at the mid sagittal plane.^7^ Soft tissue A-Point (A’): The deepest point on the upper lip determined by a line joining SN with the upper vermilion border.^7^ Upper vermilion border (UV): The point at which the upper lip tissue merges with vermilion tissue. Upper lip anterior (ULA): The most anterior point of the upper lip vermilion tissue.^9^ Lip commissure (LC): The most lateral point in the transverse plane of the lips.^10^ Stomion (St): The median point of the oral embrasure when the lips are closed.^9^ Lower lip anterior (LLA): The most anterior point of the lower lip vermilion tissue.^9^ Lower vermilion border (LV): The point at which the lower lip tissue merges with vermilion tissue. Soft tissue B-point (B’): the point at the deepest concavity between the lower vermilion border and the soft tissue pogonion.^7^ Soft tissue pogonion (Pg’): The most prominent or anterior point of the soft tissue chin in the midsagittal plane.^7^ Soft tissue menton (Me’): The most inferior point on the soft tissue chin.^7^

An independent review committee was created that included the following: plastic surgeon (n=12), sculptors (n=10), hair dressers (n=12), artists (n=16) and randomly selected individuals from the general population (n=15). The review committee was compiled with the premise that it would include those who are considered experts on facial beauty, aesthetic preference, and facial proportions. A selection of the 20 most ideal computerized sketches (10 males and 10 females) was chosen and then systematically analyzed using standardized soft tissue profile measurements. The following soft tissue landmarks were identifiable on the computerized sketches (Figure 1).

**Fig. 2:**
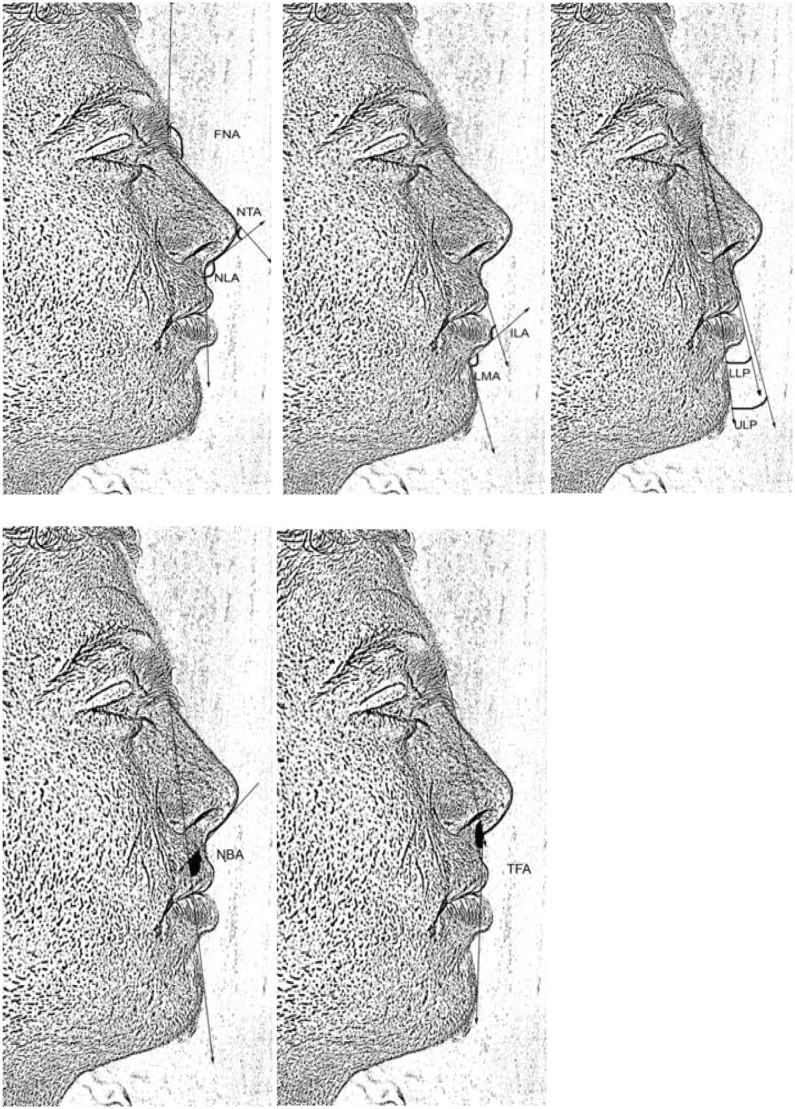
Angular measurements on soft tissue profile: a- FNA, NTA and NLA; b- ILA and LMA; c- LLP and ULP; d- NBA; e- TFA. 1. Frontonasal angle (FNA).^7^ Nasal tip angle (NTA).^8^ Nasal base angle (NBA).^8^ Nasolabial angle (NLA).^8^ Inter labial angle (ILA).^6^ Labiomental angle (LMA).^8^ Total facial angle (TFA).^9^ Upper Lip projection (ULP).^6^ Lower lip projection (LLP).^6^

**Fig. 3:**
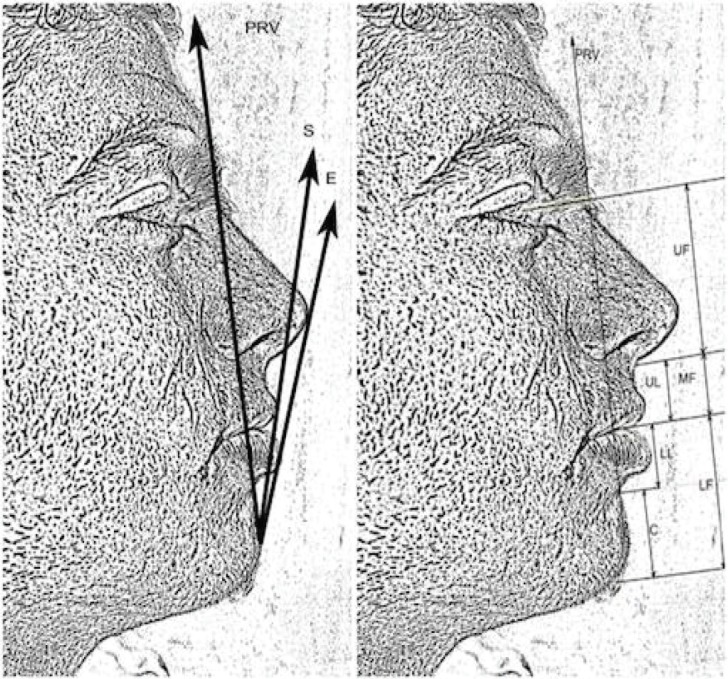
Linear measurement on soft tissue profile. a- Reference lines for measuring linear measurements: PRV line, S-line and E-line; b- facial heights according to PRV line. Upper lip anterior (ULA) to profile root vertical line (PRV) (pogonion to glabella);^2^ Lower lip anterior (LLA) to PRV. ULA to Steiner (S) line (pogonion to columella);^11^ LLA to S-line; ULA to esthetic (E)-plane (pogonion to pronasale);^12^ LLA to E-plane; Upper lip length (UL): SN to St; Lower lip length (LL): St to B’; Chin length (C): B’ to Me’; Upper facial height (UF): P to Sn; Middle facial height (MF): Sn to St; Lower facial height (LF): St to Me’

The soft tissue landmarks listed above were measured with respect to each photographic-derived sketch. Each profile was modified so that the distance between the soft tissue nasion and the subnasale (N’- Sn) was equivalent to 54 mm. A total of 21 angular and linear measurements were calculated (Figure 2 and 3). A standard protractor and millimeter ruler were used for all measurements. Angular measurements were made to the nearest 0.5 degree and linear measurements were taken to the nearest 0.5 mm and the definitions for angular measurements on soft tissue profile were as follows (Figure 2). 

Frontonasal angle (FNA): Angle formed by the intersection of lines drawn from soft tissue glabella to nasion and from nasion tangent with the superior surface of the nose.^7^ Nasal tip angle (NTA): Angle formed by the inter section of a line passing from nasion tangent to the superior surface of the nasal tip and a line passing along the greatest tangent of columella.^8^ Nasal base angle (NBA): Angle formed by the inter section of a line passing along the greatest tangent of columella and a line passing from soft tissue nasion to soft tissue pogonion.^8^ Nasolabial angle (NLA): Angle formed by the intersection of lines drawn from SN to the greatest tangent of the columella of the nose and from SN to the most anterior point on the upper lip.^8^ Inter labial angle (ILA): Angle formed by the inter section of lines drawn from A’ to UVB and from LVB to the B’ point.^6^

Labiomental angle (LMA): Angle formed by the intersection of lines drawn from LVB to B’ and from B’ to Pg. Total facial angle (TFA): Angle formed by the intersection of lines drawn from soft tissue glabella to SN and from SN to soft tissue pogonion.^9^ Upper Lip projection (ULP): Angle formed by the intersection of lines from Pg’ to nasion and from nasion to ULA.^6^ Lower lip projection (LLP): Angle formed by the intersection of lines from nasion to Pg’ and from nasion to LLA.^6^

The definitions for linear measurements were as follows (Figure 3): Upper lip anterior (ULA) to profile root vertical line (PRV) (pogonion to glabella);^2^ Lower lip anterior (LLA) to PRV. ULA to Steiner (S) line (pogonion to columella);^11^ LLA to S-line; ULA to esthetic (E)-plane (pogonion to pronasale);^12^ LLA to E-plane; Upper lip length (UL): SN to St; Lower lip length (LL): St to B’; Chin length (C): B’ to Me’; Upper facial height (UF): P to Sn; Middle facial height (MF): Sn to St; Lower facial height (LF): St to Me’.

Each angular and linear category was measured five times by the investigator, and was blindly repeated by the co-investigator. All of the measurements were averaged for a mean of each category, which was then used as the value for the study. Statistical analysis was performed on each variable including the least, greatest, mean, and standard deviation data-points. Student unpaired t-test analysis was used to compare these results from those of comparative studies in the literature. The level of statistical significance was set at p-value equal to 0.05. The results of our study were compared with the results of the Farkas^17^ and Sutter and Turley^18^ study on North American Caucasian males and females and unpaired students t-test were used to determine the differences between all of the groups.

## RESULTS

The mean, ranges, and standards deviations for all measurements are reported in Table 1 for males and Table 2 for females. In Iranian males, the NLA, ILA, and TFA all measured less than North American Caucasians (*p*<0.05). On the other hand, Iranian males ULP, LLP, ULA-E, LLA-E, ULA-S, LLA-S, ULA-PRV, and LLA-PRV all measured greater than North American Caucasians (*p*<0.05).

**Table 1:** Results in Iranian males and comparison to Caucasian males

**Variable**	**MIN**	**MAX**	**MEAN**	**SD**	***p*** ** value**
FNA	114	152	140.8±3.20	10.27	>0.05
NTA	60	94	75.6±3.39	11.52	>0.05
NBA	92	120	106.8±3.05	9.35	>0.05
NLA	80	118	97.7±3.23	10.46	<0.05
ILA	100	136	121.8±3.39	11.53	<0.05
LMA	118	144	132±3.05	9.36	>0.05
TFA	158	172	165±4.42	4.42	<0.05
ULP	5	11	7.3±1.37	1.88	<0.05
LLP	3	6	4.1±0.93	0.87	<0.05
ULA-E	2	7	4.4±1.19	1.42	<0.05
LLA-E	0	6	2.9±1.36	1.85	<0.05
ULA-S	0	4	1.4±1.12	1.26	<0.05
LLA-S	-2	3	0.8±1.32	1.75	<0.05
ULA-PRV	6	11	8.9±1.28	1.66	<0.05
LLA-PRV	2	9	6±1.50	2.26	<0.05
UL	19	30	23.1±1.72	2.99	<0.05
LL	14	21	18.3±1.45	2.11	<0.05
C	25	39	32.5±2.10	4.45	<0.05
UF	44	52	46.9±1.50	2.28	<0.05
MF	19	30	23.1±1.41	1.99	<0.05
LF	42	57	50.8±2.21	4.91	<0.05

**Table 2:** Results in Iranian females and comparison to Caucasian females

**Variable**	**MIN**	**MAX**	**MEAN**	**SD**	***p*** ** value**
FNA	144	154	149.1±1.92	3.69	<0.05
NTA	62	91	74.1±3.1	10.2	>0.05
NBA	93	124	106.5±3.43	11.6	
NLA	93	131	110.4±3.53	12.5	>0.05
ILA	98	144	123±3.75	14.1	>0.05
LMA	118	149	133.8±3.02	9.13	>0.05
TFA	151	170	161±2.24	5.03	<0.05
ULP	4	10	6.9±1.42	2.02	>0.05
LLP	0	7	3.8±1.46	2.14	>0.05
ULA-E	-8	-1	-4.3±1.58	2.5	>0.05
LLA-E	-8	2	-3.35±1.64	2.69	>0.05
ULA-S	-5	1	-1.8±1.41	2	>0.05
LLA-S	-6	3	-1.6±1.56	2.46	>0.05
ULA-PRV	4	13	9.3±1.63	2.67	>0.05
LLA-PRV	0	10	6.05±1.64	2.71	>0.05
UL	19	23	20.9±1.13	1.29	>0.05
LL	12	23	16±1.90	3.62	>0.05
C	23	32	27.5±1.69	2.88	>0.05
UF	36	46	41.1±1.64	2.72	<0.05
MF	19	23	20.9±1.13	1.29	<0.05
LF	39	49	44.1±1.81	3.28	<0.05

* NBA value for Caucasian females was not available

Iranian males UL, LL, and C are less and UF, MF and LF are greater than North American Caucasians (*p*<0.05). Other measurements including FNA, NTA, NBA, and LMA had no significant difference from the North American Caucasians (*p*>0.05). Iranian females, FNA, MF, and LF are greater than North American Caucasians, while TFA ands UF are less prominent than North American Caucasians (*p*<0.05). Other measurements including NTA, NLA, ILA, LMA, ULP, LLP, ULA-E, LLA-E, ULA-S, LLA-S, ULA-PRV, LLA-PRV, UL, LL, and C had no significant difference from the North American Caucasians (*p*>0.05).

## DISCUSSION

Understanding ethnic variations in facial skeletal and soft tissue morphology is important in performing proper pre-operative facial analysis and in formulating the aesthetic goals in particular ethnic subgroups. While studies have been carried out that demonstrate similar cross-cultural aesthetic preference among various ethnic groups, recognition of the morphological differences that exist between various ethnic groups plays an important role in the aesthetic evaluation and treatment.^11^^,^^12^

In the current study, the linear and angular facial measurements between Iranian and North American Caucasian males and females were carefully delineated. The upper lip projection and lower lip projection was significantly more prominent in Iranian males as compared with North American Caucasian males, because ULP, LLP, LLA-E, ULA-E, ULA-S, LLA-S, ULA-PRV, and LLA-PRV were greater in Iranian males. Therefore, Iranian males have lips that are fuller and more projected as compared with North American Caucasian males. This may be important in evaluating soft tissue distribution in the face and lips, which may dictate the degree to which lip augmentation or lifting should be carried out.^11^^,^^12^

UF, MF, LF were greater in Iranian males than North American Caucasian males, which indicated that Iranian males have longer face as compared with North American Caucasian males, which is important in planning orthognathic procedures and in balancing the facial thirds, especially with regard to vertical maxillary/mandibular osseous advancements and set-backs.^13^^-^^17^ NLA was greater in North American Caucasian males as compared with Iranian males suggesting that Iranian males have a more drooping nasal tip compared with North American Caucasian males. 

The morphology of the nose in Middle Easterners is a whole topic unto itself. However, a plunging nasal tip (often hyperdynamic due to depressor septi nasi muscle activity), once corrected produces a dramatic change in nasofacial balance. This will often require depressor septi nasi muscle transaction/transposition.^20^ Being sensitive to the prevailing nasal morphology in ethnic subgroups such as Iranians will help direct the surgeon to what is most in need of change.

TFA was greater in North American Caucasian males as compared with Iranian males, and therefore, Iranians males demonstrate more inclination in their general facial profile and have more convexity in their faces than North American Caucasian. This is important with regards to malar augmentation and evaluating both pre-operatively and intra-operatively, the magnitude of change in facial soft tissue redirstribution/augmentation that is required, without creating racial incongruity. Other measurements including FNA, NTA, NBA, and LMA showed no significant difference between Iranian males and North American Caucasian males.

The frontonasal area is straighter and the lower face is longer in Iranian females based on a greater. FNA, MF and LF in Iranian females compared with North American Caucasian females while the TFA and UF were greater in North American Caucasian females as compared with Iranian females indicating that Iranian females have more convex faces and the upper face is shorter. Therefore, it may be important to be especially sensitive to facial modifications in the vertical direction. 

For instance, an osseous genioplasty that increase vertical mandibular height should be very conservative so that a discrepancy between the upper, middle, and lower facial heights is not exaggerated post-operatively. Other linear and angular measurements in females did not show significant difference between our study and North American Caucasian females. In summary, the facial profile in Iranian females was more similar to North American Caucasian females than the facial profile of Iranian males as compared to North American Caucasian males. We believe that plastic surgeons must know the standards of beauty and the profile of the ideal facial soft tissue of specific ethnic subgroups. 

This familiarity will help guide the surgeon in pre-operative planning and intra-operative assessment of dynamic (“on-table”) changes that occur, so that racial incongruity is not produced. It is important, however, to stress that the treatment plan should always be dictated by individual assessment.^6^^,^^15^^-^^20^ Ethnic morphological studies such as the current paper serve as merely a guide as to the normative values of a particular ethnic population. The current study may assist the plastic surgeon for better performance of facial aesthetic and reconstructive surgery on Iranian and Middle Eastern patients.

## CONFLICT OF INTEREST

The authors declare no conflict of interest.
